# Imaging and clinical outcomes of COVID-19- vs. non-COVID-19-related cerebral venous thrombosis

**DOI:** 10.3389/fstro.2024.1396507

**Published:** 2024-12-13

**Authors:** Toska Maxhuni, Thorsten R. Doeppner, Tobias Braun, Julia Emde, Tobias Struffert, Thomas Dembek, Hagen B. Huttner, Martin B. Juenemann, Stefan T. Gerner

**Affiliations:** ^1^Department of Neurology, University Hospital Giessen, Giessen, Germany; ^2^Center for Mind, Brain and Behavior (CMBB), University of Marburg and Justus Liebig University Giessen, Marburg, Germany; ^3^Department of Neuroradiology, University Hospital Giessen, Giessen, Germany

**Keywords:** COVID-19, cerebral venous thrombosis, SARS-CoV-2, outcome, anticoagulation, thrombosis

## Abstract

**Background:**

Cerebral venous thrombosis (CVT) is a rare but serious subtype of stroke. Several studies have reported an increased incidence of CVT after either COVID-19 (CoV19) infection or vaccination; however, data on clinical characteristics, the radiological profiles, and the outcomes of these patients with CVT as the only severe symptom of a CoV19 infection or vaccination compared to patients with non-CoV19-related CVT are still scarce.

**Methods:**

We performed a retrospective monocentric study over 10 years (January 2013–December 2022) that included consecutive patients with a confirmed diagnosis of CVT based on imaging of the cerebral venous system. Patients were categorized as CoV19 CVT (either due to infection or post-vaccination) or non-CoV19 CVT and compared regarding demographics, risk factors, clinical characteristics, and imaging findings as well as outcome (at discharge, at 6 months, and last follow-up). Furthermore, sub-analyses were performed to compare CoV19-infection-related-CVT and CoV19-vaccination-related-CVT patients.

**Results:**

Overall, 122 patients with suspected CVT were identified. After excluding patients with missing data (*n* = 20) or missing imaging of the cerebral venous system (*n* = 31), 71 patients with confirmed CVT remained for the final analyses. Of those, 11 patients had CoV19 CVT (infection *n* = 3, vaccination *n* = 8), and 60 patients had non-CoV19-CVT. There were no differences regarding median age (CoV19: 40 [IQR: 22–70] vs. non-CoV19: 41 [IQR:27–64]) or percentage of female sex among both groups. A lower rate of CVT risk factors was observed in the CoV19 group but without significant differences. No patient with CoV19 CVT displayed impaired consciousness on presentation, and only 30% had focal neurological deficits compared to 51.7% in the control group. The rate of CVT-related intracranial hemorrhage and venous infarcts were 27.3% and 9.1%, respectively, in the CoV19 group and 30% and 16.7%, respectively, in the non-CoV19 group. The mortality rate at discharge was 9.1% in the CoV19-CVT group vs. 3.3% in the non-CoV19-CVT group, without differences in functional outcomes during the follow-up period. Sub-analyses comparing CoV19-infection-related CVT vs. CoV19-vaccination-related CVT patients revealed no significant differences in clinical, imaging, or treatment characteristics.

**Conclusion:**

In this monocentric study, there was no signal for a worse severity of CoV19 CVT compared to non-CoV19 CVT regarding clinical characteristics, imaging profile, or outcomes in patients with CVT only. Larger observational data with sophisticated workups of CVT patients are needed to confirm our results.

## Introduction

Cerebral venous thrombosis (CVT) is a rare subtype of stroke accounting for 0.5%−2% of all stroke cases and is characterized by blood clot formation within dural sinuses and cerebral veins (Wang et al., 2023). Several etiologies contribute to CVT development; the emergence of the novel coronavirus-19 disease (COVID-19 [CoV19]) has raised concerns about a potential association with increased risk for CVT due to both an active infection and after CoV19 vaccination (Schulz et al., 2021; Kallel et al., 2022).

CoV19, caused by severe acute respiratory syndrome coronavirus 2 (SARS-CoV 2), is associated with a variety of neurological complications, of which CVT gained substantial attention. Previous studies suggested that CoV19 infection triggers a detrimental prothrombotic cascade, including immune dysregulation, endothelial dysfunction, and hypercoagulatory state (Jose and Manuel, 2020). In line with these studies, an increased rate of CVT was observed in these patients up to 90 days after the initial infection (Schulz et al., 2021; Scutelnic et al., 2024). A very similar association was reported in patients after vaccination against CoV19 (Schulz et al., 2021; Jose and Manuel, 2020; Greistorfer and Jud, 2023; Scutelnic et al., 2022, 2023). Although the pathophysiological mechanism is not completely understood, vaccine-induced immune responses linked with thrombopenia are involved in vaccinated patients developing CVT (Schulz et al., 2021; McCullough-Hicks et al., 2022; Roytenberg et al., 2023; Greinacher et al., 2022; Braun et al., 2021).

CVT is most accurately diagnosed using advanced neuroimaging techniques, if available. Computed tomography (CT) venography and magnetic resonance (MR) venography are widely recognized as the standard approaches for detecting thrombi and assessing the extent of sinus involvement. MR venography, especially sequences such as T2-SPACE, offers high accuracy in detecting CVT even without contrast, making it a valuable tool in settings where contrast agents are contraindicated (Linn and Brückmann, 2010; Patel et al., 2016). These imaging modalities are essential for diagnosing the presence and extent of CVT to guide further treatment.

So far, several studies have reported higher mortality and incidence of CoV19-associated CVT (Tu et al., 2022), but an in-depth comparison of clinical profiles and long-term follow-up, including clinical and radiological outcomes among CoV19- and non-CoV19-CVTs, are still scarce, not completely available (Kallel et al., 2022; Scutelnic et al., 2024; Roy-Gash et al., 2020; van de Munckhof et al., 2022), or primarily focus on CVTs occurring due to the adenoviral COVID-19 vaccination (Schulz et al., 2021; Scutelnic et al., 2022, 2023). Therefore, this monocentric study aims to address this gap by exploring both infection- and vaccination-related CVT and investigating the severity, clinical manifestations, radiological characteristics, and long-term outcomes of CVT by comparing CoV19- and non-CoV19 patients.

## Methods

### Study design

This retrospective monocentric study was conducted at the Department of Neurology, University Hospital Giessen, Germany. The study aimed to assess and compare patients with CVT over 10 years (January 2013–December 2022). We enrolled patients admitted primarily (a) with a confirmed diagnosis of CVT by advanced brain imaging based on either CT or MR venography to our department of neurology and (b) without relevant other CoV19-associated complications necessitating acute treatment. Patients younger than age 18 and those lacking a verified CVT diagnosis were excluded. Written consent was obtained from all patients or their relatives, as appropriate. This study was part of the retrospective Giessen Stroke registry (GIST; prospective part at clinicaltrials.gov ID: NCT05295862), which was approved by the local ethics committee of the Faculty of Medicine (FB11), Justus-Liebig University Giessen (decision reference: AZ 220/21).

CoV19 CVT was scored if the CoV19 infection or prior vaccination was identified as the primary cause of CVT by the treating physician according to the following criteria. CoV19-associated CVT was scored if (a) a CoV19 infection was detected by an antigen screening test and proved by polymerase chain reaction (PCR) testing, (b) the time interval between infection and first occurrence of CVT symptoms was < 60 days, and (c) the treating physician linked CVT to the former CoV19 infection mainly due to the absence of underlying other known high-risk thromboembolic risk factors necessitating oral anticoagulation. Other concurrent upper respiratory tract infections were excluded by multiplex PCR for respiratory viruses. Vaccination-associated CVT was scored if (1) a vaccination against CoV19 was performed, (2) the time interval between vaccination and the first occurrence of CVT symptoms was between 14 and 60 days, and (3a) the patient had no other known underlying high-risk thromboembolic risk factors or (3b) at least possible vaccine-induced thrombotic thrombocytopenia (VITT). The VITT criteria were adapted from Pavord et al. (2021).

All the CoV19-CVT patients were admitted primarily for CVT and had no other symptoms necessitating hospitalization. Non-CoV19 CVT cases were defined as CVT cases not timely or causatively linked to a CoV19 infection or vaccination. In cases with several risk factors and the presence of a CoV19 infection or vaccination, these patients were grouped into the CoV19-CVT cohort.

### Clinical parameters

By electronic chart review, we collected individual parameters of CVT patients, including demographic data (age and sex), thrombotic risk factors (e.g., active smoking or acquired or genetic coagulopathy), procoagulatory drug intake (such as hormone therapy or birth control), clinical presentation on admission assessed using the Glasgow Coma Scale (GCS), National Institutes of Health Stroke Scale (NIHSS), and treatment characteristics, with a focus on acute management and antithrombotic treatment regime. Imaging on admission was assessed by review of CT or MR angiography to document the location of the thrombus, the number of affected sinuses, and the concomitant edema or intracerebral hemorrhage (ICH): ICH volume was estimated by the established ABC/2 formula (Huttner et al., 2006). Furthermore, electroencephalographic (EEG) changes were recorded in patients. Laboratory workup included platelet count, D-Dimers, C-reactive protein and standard coagulation parameters.

In CoV19-CVT group, we further documented the time from CoV19 symptom onset to hospital admission, the type and dose of vaccine, platelet factor 4 (PF4) enzyme-linked immunosorbent assay (ELISA) test (vaccine-associated CVT) or PCR test (infection-associated CVT) results, and specific treatments such as Fondaparinux (Argatra) and immunoglobulins for vaccine-related CVT or antiviral treatment for infection-related CVT (Greinacher et al., 2022; Marchetti et al., 2022; Nazy et al., 2021; Gabarin et al., 2022).

### Follow-up parameters

Patient outcomes were assessed at discharge, 6 months, and last follow-up in our outpatient clinic using the NIHSS and the modified Rankin Scale (mRS). Furthermore, we assessed the recanalization rate of the thrombosed sinus if adequate imaging was available at the time of presentation. The prediction of the rate of 30-day case fatality was assessed by the Cerebral Venous Thrombosis Grading Scale (CVT-GS) at discharge, which was categorized as mild (0–2 points), moderate (3–7 points), or severe (8–13 points), as described elsewhere (Barboza et al., 2018).

### Outcome measures

The primary endpoint was the rate of excellent functional outcome (i.e., mRS score of 0 or 1) at last follow-up. Exploratory endpoints included a clinical and radiologic profile on admission, the outcome at discharge using CVT-GS and mRS scores at 6 months as well as at last follow-up, and the rate of complete recanalization CT angiography or MR angiography at 6 months and last follow-up.

### Statistical analysis

All data analyses were performed by SPSS (IBM SPSS Statistics 28.0), and illustrations were created using Adobe Illustrator (Adobe, Adobe Illustrator 2023). Patients were dichotomized into two groups: CoV19-CVT patients and non-CoV19-CVT patients. Comparisons were undertaken using the chi-squared test for nominal and the Mann–Whitney *U* test for non-normally distributed data. Categorical variables are presented as the number and the percentage (%), and the quantitative variable was presented as the median with the interquartile range (IQR). Continuous data were presented as means with the standardized difference and were analyzed using Student's *t*-test. The affected sinuses' location and distribution of functional outcomes on the mRS were graphically illustrated. A prespecified sub-analysis was conducted for intra-group comparisons of patients with CoV19-CVT. Therefore, analyses regarding clinical, radiological, and outcome parameters were conducted to compare CoV19-infection-related CVT vs. CoV19-vaccination-related CVT.

## Results

**Figure 1:**
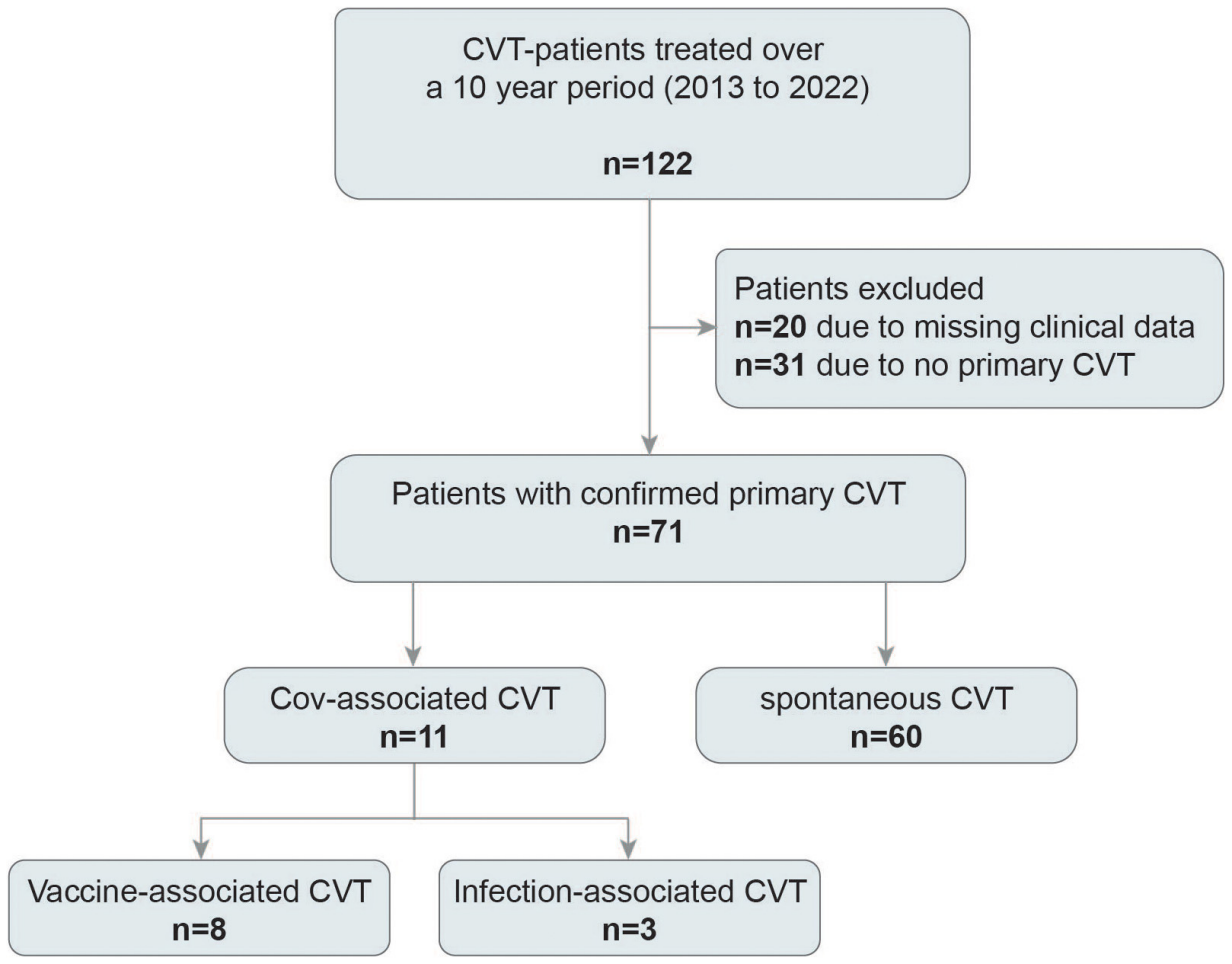
Flowchart of included patients. Over 10 years, 122 patients with suspected cerebral venous thrombosis (CVT) were identified, of those, 71 remained for final analyses.

Over the 10 years, 122 patients with suspected CVT were identified. Among those, 51 patients were excluded (*n* = 31 due to CVT not confirmed by venography and *n* = 20 due to missing data). Therefore, 71 patients with imaging-confirmed CVT remained for final analyses, and of those, 11 patients were diagnosed with CoV19-CVT, as shown in Figure 1.

### Demographic information and risk factors

Demographic information, risk factors, and clinical presentation comparing Cov19-CVT and non-CoV19-CVT patients are displayed in Table 1. The median age was 40 years in the CoV19-CVT group and 41 in non-CoV19-CVT group. Female patients constituted 36.4% of the CoV19 cohort and 65% of the non-CoV19 cohort (*p* = 0.09).

**Table 1:** Characteristics of included patients.

**Patients with CVT (*n* = 71)**	**CoV19–CVT (*n* = 11)**	**Non–CoV19–CVT (*n* = 60)**	***P* value**
Age, y; median (IQR)	40 (22–70)	41 (27–64)	0.97
Female sex; *n* (%)	4 (36.4)	39 (65.0)	0.09
**Risk factors**			
History of VTE; *n* (%)	0 (0)	3 (5.0)	>0.99
Active smoking; *n* (%)	0 (0)	8 (13.3)	0.59
Procoagulatory drugs; *n* (%)	0 (0)	18 (30)	0.05
Pregnancy or postpartum; *n* (%)	1 (9.1)	2 (3.3)	0.40
Hereditary coagulopathy; *n* (%)	4 (36.3)	27 (45)	>0.99
Infection or malignancy; *n* (%)	0 (0)	10 (16.7)	0.34
VITT; *n* (%)	8 (72.7)	0 (0.0)	>0.99
**Clinical presentation on admission**			
Headache; *n* (%)	9 (81.8)	40 (67.8)	0.48
Visual impairment; *n* (%)	1 (9.1)	14 (23.3)	0.44
Focal deficit; *n* (%)	3 (27.3)	31 (51.7)	0.122
NIHSS; median (IQR)	0 (0–2)	1 (0–4)	0.11
No deficit; *n* (%)	7 (63.6)	29 (48.3)	0.31
Severe (≥5); *n* (%)	0 (0)	11 (18.3)	0.20
mRS; median (IQR)	0 (0–1)	1 (0–2)	0.15
Seizures; *n* (%)	3 (27.2)	20 (33.3)	>0.99
Impaired consciousness; *n* (%)	0 (0)	10 (16.7)	0.34
GCS; median (IQR)	15 (15–15)	15 (15–15)	0.17
**Imaging on admission**			
CT-venography; *n* (%)	8 (72.7)	45 (75)	1.00
MRI-venography; *n* (%)	8 (81.8)	45 (75)	1.00
Venous infarct; *n* (%)	1 (9.1)	10 (16.7)	1.00
Intracranial hemorrhage; *n* (%)	3 (27.3)	18 (30)	1.00
ICH-volume, mL; median (IQR)	1.2 (0.5–1.2)	0.6 (0.2–1.4)	0.76
More than 1 sinus affected; *n* (%)	11 (100)	44 (73.3)	0.10
**EEG findings**			
EEG performed; *n* (%)	3 (27.2)	32 (53.3)	0.17
Any EEG pathology; *n* (%)	2/3 (66.7)	21/32 (56.2)	>0.99
Epileptic potential; *n* (%)	1 (9.1)	1 (3.1)	0.29
Focal lesion; *n* (%)	1 (9.1)	20 (62.5)	0.08
**Laboratory measurements**			
D-Dimer performed; *n* (%)	10 (90.9)	41 (68.3)	0.16
D-Dimer count, mg/L; mean (SD)	2.1 (1.3–6.1)	1.2 (0.6–2.2)	0.10
D-Dimer positive; *n* (%)	9/10 (90)	33/41 (80.5)	0.67
Platelet count,/μL; median (IQR)	176 (78–269)	254 (210–321)	0.018
C-reactive protein, mg/L; median (IQR)	4.1 (3.2–9.4)	10.9 (2.9–32.8)	0.14
**Acute treatment**			
Anticoagulation treatment; *n* (%)	11 (100)	60 (100)	>0.99
LMH; *n* (%)	7 (63.6)	35 (57.6)	>0.99
UFH; *n* (%)	4 (36.3)	25 (42.4)	>0.99
Antiseizure medication; *n* (%)	3 (27.2)	21 (35)	>0.99
Hemicraniectomy; *n* (%)	1 (9.1)	1 (1.7)	0.29
**Outcomes at discharge**			
Length of hospital stay, d; median (IQR)	11 (7–16)	13 (9–18)	0.42
Death; *n* (%)	1 (9.1)	2 (3.3)	0.40
Modified Rankin score; median (IQR)	0 (0–3)	0 (0–1)	0.67
NIHSS; median (IQR)	0 (0–2.25)	0 (0–1)	0.32
**CVT-GS**			
Mild (0–2P); *n* (%)	8 (72.7)	51 (85)	0.65
Moderate (3–7P); *n* (%)	2 (18.1)	7 (11.7)	0.61
Severe (8–13P); *n* (%)	1 (9.1)	2 (3.3)	0.40
**Discharge destination**			
Home; *n* (%)	7 (63.6)	42 (70)	>0.99
Rehabilitation; *n* (%)	2 (18.1)	15 (25)	>0.99
**Outcomes at 6 months**			
Complete recanalization; *n* (%)	1/5 (20)	4/18 (22.2)	>0.99
mRS; median (IQR); *n* = 41	0 (0–0)	0 (0–0)	0.84
NIHSS; median (IQR); *n* = 41	0 (0–0)	0 (0–0)	0.93
No deficit; *n* (%)	6/6 (100)	34/35 (97.1)	>0.99
**Outcomes at 12 months**			
Complete recanalization; *n* (%)	4/8 (50)	8/28 (28.6)	0.40
mRS; median (IQR); *n* = 53	0 (0–0)	0 (0–0)	0.71
NIHSS; median (IQR); *n* = 52	0 (0–0)	0 (0–0)	0.63

Comparison of CoV19- vs. non-CoV19-CVT patients regarding demographic data, prior medical history, including prothrombotic risk factors, clinical presentation, imaging profile, treatment and outcomes. Values are presented either as number (percentage) or median (interquartile range).

CoV19, COVID-19; CVT, cerebral venous thrombosis; VTE, venous thromboembolism; VITT, vaccine-induced immune thrombotic thrombocytopenia; EEG, electroencephalography; LMH, low-molecular-weight heparin; UFH, unfractionated heparin.

Risk factors for CVT were more frequently observed in the non-CoV19 group. In the CoV19-CVT group, the most frequent, in descending order, were VITT (72.7%), hereditary coagulopathy (36.3%), and pregnancy (9.1%); in the non-CoV19-CVT group, they were hereditary coagulopathy (45%), procoagulatory drugs (30%), infection or malignancy (16.7%), smoking (13.3%), history of thrombosis (5%) and pregnancy or postpartum (3.3%; Table 1).

### Clinical presentation and diagnostic including laboratory findings

Headache was the predominant symptom in both groups (81.8% in CoV19-CVT and 67.8% in non-CoV19-CVT patients). CoV19-CVT patients had less frequent neurological deficits upon admission (27.3% vs. 51.7%, *p* = 0.12), and none of these patients presented with severe neurological deficits (i.e., NIHSS score >4) or impaired consciousness. The rate of initial epileptic seizures was similar in both groups (CoV19 CVT: 27.2% vs. non-CoV19 CVT: 33.3%). On admission, none of the CoV19-CVT group and 10 patients (16.7%) in the non-CoV19-CVT group presented with impaired consciousness.

**Figure 2:**
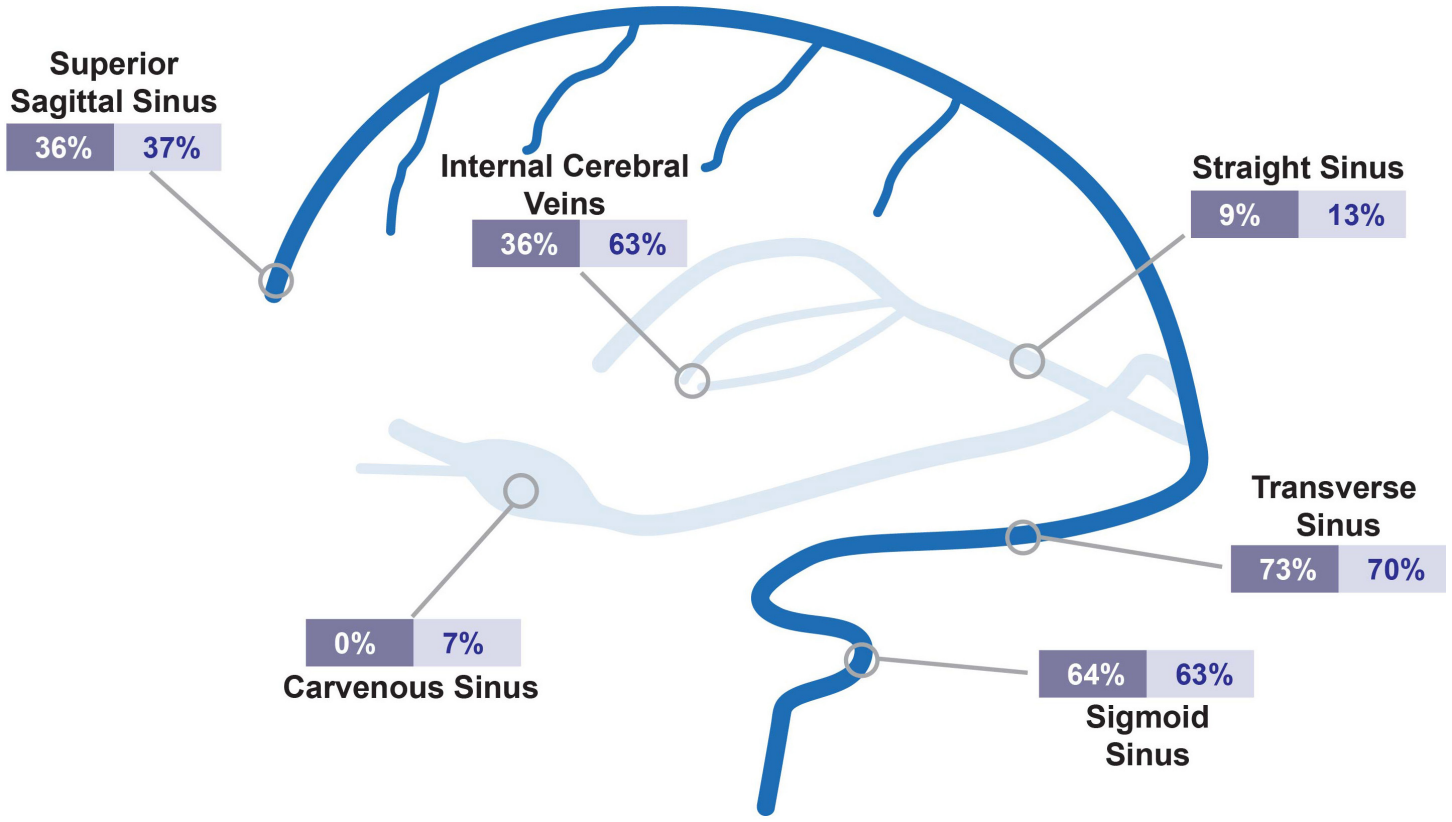
Distribution of affected sinuses in CVT-patients. Illustrated are affected sinuses or cerebral veins. Relative percentages are shown for CoV19 CVT (dark blue) and non-CoV19 CVT (bright blue). CVT, cerebral venous thrombosis; CoV19, COVID-19.

On initial imaging, the cerebral infarction rate and intracranial hemorrhage were 9.1% and 27.3%, respectively, for the CoV19-CVT patients and 16.7% and 30%, respectively, for non-CoV19-CVT patients (Table 1). All CoV19 patients (100%) and 44 (73.3%) control patients had more than one sinus affected by thrombosis. The distribution of affected sinuses is illustrated in Figure 2. In essence, there was no significant difference in the distribution of thrombosed sinuses; the most frequent sinuses affected were the transverse sinus (CoV19 CVT: 73%; non-CoV19 CVT: 70%) and the sigmoid sinus (64% vs. 63%, respectively). EEG was performed in 3 patients (27.2%) of the CoV19-CVT group and 32 (53.3%) patients in the control group (*p* = 0.17). Of those, 2 (66.7%) and 18 (56.2%) of patients, respectively, had pathological changes on their EEG recordings. Among the pathological EEG findings, in both groups, only one patient had epileptic typical potentials (9.1% vs. 3.1%). However, a difference was observed concerning focal abnormalities in the EEG; here, 62.5% of non-CoV19-CVT patients showed focal abnormalities in the EEG compared to only 1 CoV19-CVT patient (9.1%, *p* = 0.08) exhibiting focal abnormalities.

There was no significant difference regarding most laboratory parameters on admission as shown in Table 1. In patients with an initial D-dimer assessment (*n* = 51), D-dimer counts were pathologically elevated in 90% of CoV19-CVT and 80.5% of non-CoV19-CVT patients. Of interest, lower platelet counts were detected in CoV19-CVT-patients (median platelet count: CoV19: 176/μL vs. non-CoV19: 254/μL, *p* = 0.018; Table 1).

**Figure 3:**
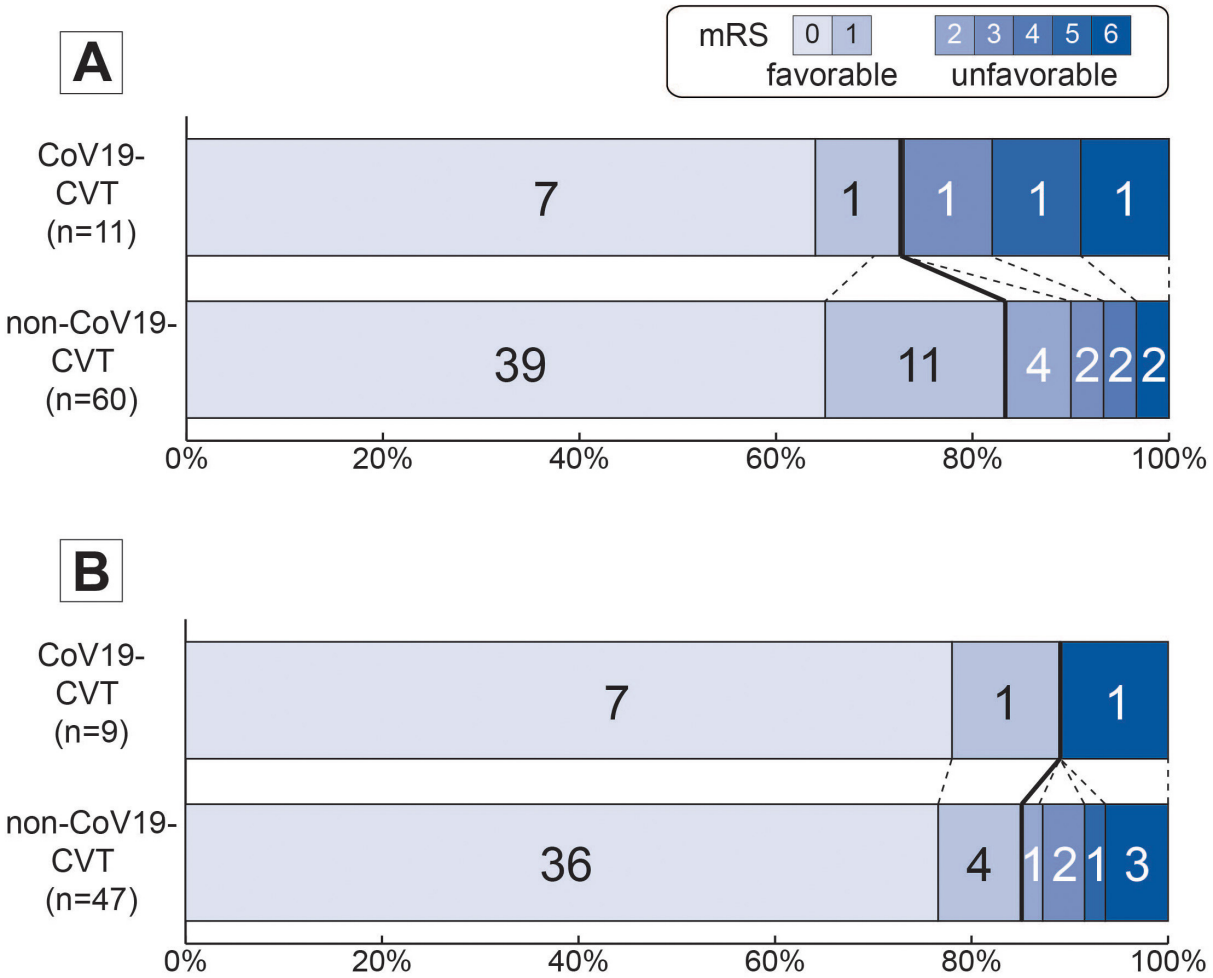
Distribution of mRS scores comparing CoV19- vs. non-CoV19-CVT patients. mRS score plots are provided for **(A)** mRS score at discharge and **(B)** at 12 months or last follow-up comparing CoV19- vs. non-CoV19 CVT. Favorable functional outcome was defined as mRS score of 0 or 1. Total numbers of patients at each mRS score is provided within the bar. mRS, modified Rankin score; CoV19, COVID-19; CVT, cerebral venous thrombosis.

All patients received acute treatment by oral anticoagulation, with low-molecular heparin being the preferred choice (60%). Anticonvulsive medication was needed in every third patient. Two patients (3.3%) of the non-CoV19-CVT group and one patient (9.1%) of the CoV19-CVT group died during hospitalization. The median mRS score at discharge was 0 for both groups (CoV19-CVT: 0 [IQR 0–3] vs. non-CoV19-CVT: 0 [0–1]; see Figure 3A). At discharge, 72.7% of CoV19-CVT patients and 85% of non-CoV19-CVT patients had a mild CVT-GS score, translating to a predicted low 30-day fatality rate.

### Outcomes at 6 months and last follow-up

At the 6-month follow-up, just 1 of 5 CoV19-CVT patients (20%) and 4 of all 18 (22.2%) of the non-CoV19-CVT patients had a complete thrombus recanalization. The median mRS score was 0 for both groups (IQR [0–0] vs. [0–0]).

At last follow-up (average 12 months), excellent functional outcome (i.e., mRS score of 0–1) was achieved by all CoV19-CVT patients (100%) and 85.1% of non-CoV19-CVT patients (illustrated in Figure 3B). The median mRS score was 0 for both groups (IQR [0–0] vs. [0–0]).

50% (4/8) among patients, 50% of those with CoV-CVT and 28.6% (8/28, *p* = 0.4) of those with non-CoV19-CVT had a complete thrombus recanalization.

### Specific differences among cov19-CVT patients (infection vs. vaccination)

Specific characteristics of CoV19-CVT patients are presented in Table 2. In eight patients, CVT was assessed as vaccination-related, with a median of 14 days between vaccination and hospital admission. Three patients were vaccinated with ChAdOx1 nCov19 vaccine (AstraZeneca), had a positive PF4-ELISA test, and received additional therapy with intravenous immunoglobulin (IVIG) at a dose of 1 g/kg body weight for 2 days as well as non-heparin anticoagulation (Argatroban). The third patient died during the hospitalization on the seventh day after admission due to a cerebral hemorrhage and the subsequent increase in edema formation (Braun et al., 2021). Patients 4–7 received the BNT162b2 vaccine (BioNTech), and their PF4-ELISA and heparin-induced thrombocytopenia−2 (HIT-2) tests were negative. They were treated with heparin anticoagulation and no IVIG. The last patient was admitted 30 days after the second dose of the vaccine (Chinese vaccine; not further specified). PF4-ELISA and HIT-2 tests were negative, and the patient received heparin anticoagulation and no IVIG.

**Table 2:** Specific differences among CoV19-SVT patients (CoV19 infection vs. CoV19 vaccine).

**Vaccine associated CVT (*****n*** = **8)**							
	**Days from vaccination to hospital admission (nr, days)**	**Type of vaccine**	**Which dose of vaccine**	**PF4-ELISA test**	**HIT-2 (yes or no)**	**Therapy with Fondaparinux (Argatra)**	**Therapy with IVIG**
Patient 1	14 d	AstraZeneca	First	Positive	No	Yes	Yes
Patient 2	22 d	AstraZeneca	First	Positive	No	Yes	Yes
Patient 3	10 d	AstraZeneca	First	Positive	No	Yes	Yes
Patient 4	44 d	BioNTech	Second	Negative	No	No	No
Patient 5	14 d	BioNTech	Second	Negative	No	No	No
Patient 6	13 d	BioNTech	Second	Negative	No	No	No
Patient 7	14 d	BioNTech	Second	Negative	No	No	No
Patient 8	30 d	Other	Second	Negative	No	No	No
**Infection associated CVT (*****n*** = **3)**							
	**Days from infection to symptom onset**	**Days from symptom onset to admission**	**PCR-Test at admission**	**Ct-value at admission**	**Virus variant**	**Covid-19 severity**	**Antiviral treatment**
Patient 1	0 d	12 d	Negative	>35	Unknown	Mild	No
Patient 2	7 d	60 d	Negative	>35	Unknown	Mild	No
Patient 3	0 d	0 d	Positive	>35	Unknown	Moderate	No

Specific information on CoV19-infection- vs. CoV19-vaccine-related-CVT patients regarding type of vaccine, laboratory testing and therapy.

CoV19, COVID-19; CVT, cerebral venous thrombosis; PF4, platelet factor 4; IVIG, intravenous immunoglobulin; PCR, polymerase chain reaction.

In patients with CoV19-infection-related CVT (Table 2), the first patient was admitted to the emergency department 12 days after symptom onset. Their PCR test was negative; the infection course was mild, and therefore, the patient was not treated with antiviral therapy. The second patient was admitted 60 days after symptom onset; however, he had had a headache since the second day of infection. The PCR test was negative; the course of infection was mild as well, and he was not treated with antiviral therapy. The third patient was admitted the same day as symptom onset (d0), with a positive PCR test with CT value of >35. The infection's course was moderate, and he was not treated with antiviral therapy. For all three patients, the virus variant was unknown.

The demographic and imaging profiles of both CoV19-CVT entities were not different (Table 3); however, long-term follow-up revealed no patient achieving complete recanalization in the CoV19-infection group (infection: 0/3 vs. vaccination: 4/5).

**Table 3:** Clinical and diagnostic profile of CoV19-infection- vs. vaccination-related CVT.

**Characteristics**	**COVID-19 infection; *N* = 3**	**COVID-19 vaccination; *N* = 8**	***p*-value**
Age, median (IQR)	40 (23–75)	46 (20–72)	0.73
Female sex, *n* (%)	0 (0)	4 (50)	0.24
NIHSS on admission, median (IQR)	2 (0–2)	0 (0–0)	0.09
Headache, *n* (%)	2 (66.7)	7 (87.5)	>0.99
Focal deficit, *n* (%)	2 (66.7)	1 (12.5)	0.15
Epileptic seizure, *n* (%)	1 (33.3)	2 (25.0)	>0.99
**Diagnostic**			
CT-venography; *n* (%)	2 (66.7)	6 (75.0)	>0.99
MRI-venography; *n* (%)	3 (100.0)	6 (75.0)	>0.99
Venous infarct; *n* (%)	0 (0.0)	1 (12.5)	>0.99
Intracranial hemorrhage; *n* (%)	0 (0.0)	3 (37.5)	0.49
D-Dimer positive, *n* (%)	3 (100.0)	7 (87.5)	>0.99
**Discharge**			
mRS, median (IQR)	1 (0–1)	0 (0–0)	0.21
Death, *n* (%)	0 (0)	1 (12.5)	>0.99
**Outcome at 6 months**			
mRS, median (IQR)	0 (0–0)	0 (0–0)	0.16
Complete recanalization, *n* (%)	0/2 (0.0)	1/4 (14.3)	>0.99
**Outcome at last follow-up**			
mRS, median (IQR)	0 (0–0)	0 (0–0)	0.20
Complete recanalization, *n* (%)	0/3 (0)	4/5 (80)	0.14

Specific information on CoV19-infection vs. CoV19-vaccine related CVT patients regarding type of vaccine, laboratory testing and therapy.

CoV19, COVID-19; CVT, cerebral venous thrombosis; PF4 indicates platelet factor 4; IVIG, intravenous immunoglobulin; PCR, polymerase chain reaction.

## Discussion

In this retrospective study, we are, to our knowledge, the first to describe granular clinical data, imaging profiles, and long-term outcomes of patients with CoV19-CVT and no other symptoms indicating hospitalization. There was no statistical trend toward higher severity of 11 patients with CoV19-CVT compared to a control group of 60 non-CoV19-CVT patients. The overall clinical course was mild with no relevant clinical impairment during the follow-up period of at least 12 months. Several aspects deserve special attention.

First, despite reports of increased incidence of CVT in either CoV19 infection or after vaccination, there are still uncertainties about the severity and clinical course of CoV19 CVT (Scutelnic et al., 2024; McCullough-Hicks et al., 2022; Tu et al., 2022; Helms et al., 2020). In this monocentric experience, we identified 11 cases of CoV19 CVT, with overall benign clinical and radiological profiles except for one case. In essence, we observed no significant differences compared to non-CoV19 CVT but, rather, detected signals of a less severe manifestation of CVT in the CoV19 group compared to the non-CoV19 group. This observation is reflected by the relatively benign hospital course and the outcome-relevant complication rates – such as additional intracerebral hemorrhage or stroke due to CVT—in CoV19 patients. Regarding laboratory profiles, we detected lower platelet counts in the CoV19-CVT group, which is mainly explained by reduced platelet counts in patients with VITT-associated CVT (*n* = 3, all with platelet counts below 50/μL). Furthermore, the clinical presentation with focal neurological deficits was rare in CoV19-CVT patients; most had headache as the single clinical symptom. The largest analysis of multinational CoV19-CVT cases reported a higher rate of neurologic deficits (up to 50%) and intracranial lesions (up to one third) but were comparable to the control group of non-CoV19-CVT patients (Scutelnic et al., 2024). In conclusion, absent other CoV19-associated complications, the clinical course of CoV19 CVT does not seem more severe than CVT attributed to other causes. However, it should be noted that underlying comorbidities and prothrombotic risk factors—such as cancer, genetic clotting disorders, or hormone therapy—might also influence clinical outcomes in both CoV19-related and non-CoV19-related CVT cases. Although our study attempted to account for these factors, the small sample size limits our ability to fully assess their impact. Future studies with larger, well-controlled samples are essential to further investigate these confounding variables and clarify their role in the clinical progression and outcomes of CVT in CoV19 patients.

Second, overall favorable long-term outcomes were observed in CoV19-CVT patients, with a significant proportion achieving complete recanalization during follow-up. The evaluation of recanalization rates in CoV19 CVT reveals a notably higher tendency toward vascular restoration. One plausible explanation for this is the relatively lower thromboembolic risk profile observed in CoV19 patients upon admission. Therefore, the favorable recanalization rates can be attributed to the absence of a preexisting thromboembolic burden, facilitating a smoother and more efficient vascular recovery process.

Third, an intriguing observation emerged from our comparative sub-analysis, highlighting a more favorable tendency toward complete recanalization at the 12-month follow-up in the CoV19-vaccination-related-CVT group, in contrast to the CoV19-infection-related-CVT group, where none of the three patients demonstrated complete recanalization. The underlying pathophysiology and the variation in immune responses between CoV19 infection and vaccination, which can influence thrombotic events and recanalization, may explain this disparity (Del Valle et al., 2020; Tay et al., 2020; Sahin et al., 2020). During a CoV19 infection, the virus triggers an inflammatory cascade characterized by elevated proinflammatory cytokines and often an exaggerated immune reaction (Del Valle et al., 2020; Tay et al., 2020). In contrast, CoV19 vaccines are designed to induce a more controlled and specific immune response. They stimulate the immune system to generate targeted antibodies and memory cells (Sahin et al., 2020). Unfortunately, the small sample size of this study did not allow for valuable sub-analyses of different vaccination regimes or VITT gradings as provided by larger published studies (Schulz et al., 2021; Scutelnic et al., 2022, 2023). The discrepancy in immune responses evoked by CoV19 infection and vaccination has significant implications for thrombotic events like CVT. A vaccination's ability to induce a precise and controlled immune response underscores its potential in preventing or mitigating thrombotic events and encouraging favorable vascular recovery. However, the observed higher recanalization rates in vaccinated CoV19-CVT cases compared to infection-related CoV19-CVT cases should be interpreted with caution. The small sample size and the lack of standardized criteria for assessing recanalization are limiting factors. Future studies should apply uniform recanalization criteria to improve the results' reliability and comparability.

This study has several limitations mainly due to its retrospective monocentric design. A key limitation of this study is the small sample size, particularly in the CoV19-related-CVT subgroup, which reduces our findings' statistical power and external validity. Additionally, as a single-center study with a retrospective design, selection and confounding biases may potentially exist, and the results may not be generalizable to broader populations, where diagnostic and treatment protocols may vary. Future multicenter studies with strict follow-up protocols are warranted to validate these findings and enhance external validity. Furthermore, a longer and extensive follow-up of CVT patients addressing patient-reported and cognitive outcomes was not available and would add significant knowledge to the understanding of complications and long-term sequelae of CoV19.

## Conclusion

In this monocentric study, patients with CoV19 CVT had a similar severity of clinical and radiological parameters, as well as outcomes, compared to patients with non-CoV19 CVT. Overall, the observed clinical course of CoV19 CVT was relatively mild, which needs to be confirmed in larger observational studies.

## Data Availability

The original contributions presented in the study are included in the article, further inquiries can be directed to the corresponding author.
